# Evaluation of an AI-Based Clinical Decision Support System for Perioperative Care of Older Patients: Ethical Analysis of Focus Groups With Older Adults

**DOI:** 10.2196/71568

**Published:** 2025-10-17

**Authors:** Nina Parchmann, Marcin Orzechowski, Simone Brefka, Florian Steger

**Affiliations:** 1 Institute of the History, Philosophy and Ethics of Medicine, Ulm University, Oberberghof 7, Ulm, 89081, Germany, 49 73150-39914; 2 Institute for Geriatric Research at AGAPLESION Bethesda Ulm, Ulm University Medical Center, Ulm, Germany

**Keywords:** medical ethics, older patient care, artificial intelligence, AI, clinical decision support system, focus groups

## Abstract

**Background:**

The development and introduction of an artificial intelligence (AI)–based clinical decision support system (CDSS) in surgical departments as part of the “Supporting Surgery with Geriatric Co-management and AI” project addresses the challenges of an increasingly aging population. The system enables digital comanagement of older patients by providing evidence-based evaluations of their health status, along with corresponding medical recommendations, with the aim of improving their perioperative care.

**Objective:**

The use of an AI-based CDSS in patient care raises ethical challenges. Gathering the opinions, expectations, and concerns of older adults (as potential patients) regarding the CDSS enables the identification of ethical opportunities, concerns, and limitations associated with implementing such a system in hospitals.

**Methods:**

We conducted 5 focus groups with participants aged 65 years or older. The transcripts were evaluated using qualitative content analysis and ethically analyzed. Categories were inductively generated, followed by a thematic classification of participants’ statements. We found that technical understanding did not influence the older adults’ opinions.

**Results:**

Ethical opportunities and concerns were identified. On the one hand, diagnosis and treatment could be accelerated, the patient-AI-physician interaction could enhance medical treatment, and the coordination of hospital processes could be improved. On the other hand, the quality of the CDSS depends on an adequate data foundation and robust cybersecurity. Potential risks included habituation effects, loss of a second medical opinion, and illness severity influencing patients’ attitude toward medical recommendations. The risk of overdiagnosis and overtreatment was discussed controversially, and treatment options could be influenced by interests and finances. Additional concerns included challenges with time savings, potential declines in medical skills, and effects on the length of hospital stay.

**Conclusions:**

To address the ethical challenges, we recommend allocating sufficient time for use of the CDSS and emphasizing individualized review of the CDSS results. Furthermore, we suggest limiting private financial sponsorship.

## Introduction

### Background

The global population is aging. A combination of increasing life expectancy [1] and enduring demographic shifts [2] has led to an increase in people aged 65 years or older [3]. Older patients face an increased risk of losing physical function and independence during hospitalization [4]. Comanagement of older patients, defined as a process of joint decision-making between surgical staff and a geriatrician, can improve outcomes such as mortality, length of hospital stay, and perioperative complications [5,6]. The rising prevalence of age-related conditions, including cognitive impairment and multimorbidity [7,8], further increases the demand for individualized, patient-centered treatment and resource-intensive medical care at a time when resources are already limited [2]. In parallel, the medical application of artificial intelligence (AI) is expanding, with the potential to enhance personalized medicine, patient care, prediction, and diagnostics while improving accuracy and efficiency and enabling earlier intervention [9].

A systematic review found the use of clinical decision support systems (CDSSs) to be effective with regard to both process- and patient-related outcomes [10].

The research project “Supporting Surgery with Geriatric Comanagement and AI” addresses the growing challenges in the health care system by developing and introducing an AI-based CDSS for perioperative care of older patients and subsequent care planning [11]. This system automatically processes and analyzes patient data to provide an evidence-based evaluation of the individual’s health condition and corresponding medical recommendations. The CDSS aims to improve patient care and support medical professionals in surgical wards. However, the introduction of the CDSS also raises ethical challenges [12].

The benefits of medical AI systems are offset by concerns regarding accountability and transparency, biases in training data that may compromise safety, inequities in access, and data security risks [9]. Considering that older adults represent a patient group considered vulnerable and requiring particular protection, addressing AI-related ethical challenges to safeguard patient integrity is of major importance.

The application of large language models in hospitals as a means of improving patient care has been scientifically analyzed across various medical fields and purposes. However, to the best of our knowledge, an ethical evaluation of their use within a CDSS, particularly in perioperative care of older patients and incorporating the perspectives of potential patients, remains lacking.

### Objectives

To explore the opinions, expectations, and concerns of potential older patients regarding the development and introduction of an AI-based CDSS in surgical wards caring for older patients, we conducted focus groups with older adults. We sought to (1) examine how the level of technical understanding influences their views and (2) identify the ethical opportunities, concerns, and limitations associated with implementing such a system in hospitals.

## Methods

### Ethical Considerations

Ethical approval was waived by the ethics committee of Ulm University, because no information on personal data, health-related data, or data on sexuality of the interviewees was collected (May 16, 2024). To ensure informed consent, all participants were sent a digital copy of the consent form before the discussion and were verbally briefed on the focus group procedures. The information covered data handling, privacy protection, voluntary participation, and the right to opt out, as well as details about the project and its funding. All participants provided written informed consent to participate, and no financial incentives were offered.

### Overview

Conducting focus groups enabled us to explore the opinions, expectations, and concerns of older adults. This method allows participants to freely express their views, stimulate ideas through interaction, and provide a wide range of perspectives within each group [13,14].

Participant selection followed a deductive sampling approach. The inclusion criteria were age 65 years or older and sufficient German language skills to participate in a group discussion. Given that focus groups rely on oral conversation, we ensured that all participants met the language requirements through 3 steps. First, participants had to understand the written invitation and respond appropriately. Second, we conducted telephone conversations with participants to answer questions and schedule participation before each focus group. Third and last, before starting each focus group discussion, we verified German speaking and comprehension skills through brief conversational exchanges with all participants.

With the support of a gatekeeper, invitation letters were distributed through mailing lists targeting older adults interested in education, posted on an academic website, and further disseminated via snowball sampling. Participants were assigned to focus groups using a stratified randomization approach. First, interested individuals were divided into groups by gender. Next, an equal number of participants from each group were randomly selected and assigned to 3 different focus groups with unevaluated, mixed levels of technical understanding.

Individuals who had not yet been assigned to a group were asked to complete questionnaires assessing technical knowledge. The questionnaires were scored using a points system, and on the basis of these scores, participants were divided into 2 focus groups: one with higher technical understanding and one with lower technical understanding.

In total, 5 focus groups were conducted with 30 participants from the city of Ulm in southern Germany and surrounding areas in June and July 2024. The sessions were held face-to-face. The conversation, including technical explanations of all AI-related aspects, was held in simple language and at a slower speaking rate to accommodate age-related limitations and varying levels of technical knowledge among the participants.

The discussion guide was semistructured to allow flexible adaptation to the dynamics of each group and was organized into 5 thematic categories: general opportunities, risks and resources, accuracy and trust, data processing, patient-physician relationship, and limitations.

The discussions were recorded, transcribed, and anonymized. The quotations included in this study have been translated from German into English.

The transcripts were first analyzed using qualitative content analysis [15], followed by thematic analysis [16], while taking into account 6 analytic factors specific to focus groups [13]. Categories and subcategories were identified inductively, followed by a deductive analysis structured according to the questionnaire. Participant statements were coded and assigned to the established categories. First, 3 focus groups with mixed levels of technical understanding and the 2 groups with lower and higher technical understanding were analyzed separately, considering the 6 analytic factors [13], and then compared. As no major differences were detected between the groups with lower and higher technical understanding, all focus groups were subsequently combined for comparison and analysis.

### Ethical Principles Considered in the Analysis

After this analysis, the categories and findings were reviewed by researchers with technical expertise in medicine, medical ethics, and communication and were ethically analyzed using the principle-based approach proposed by Beauchamp and Childress [17]. This biomedical approach is widely applied as a key method for reasoning about and analyzing ethical challenges in medicine. The ethical principles considered in the analysis reflect core medical values and comprise autonomy, beneficence, nonmaleficence, and justice. The principle of autonomy refers to decision-making based on the patient’s individual preferences. Beneficence emphasizes maximizing patient benefit while balancing risks against potential benefits, and nonmaleficence aims to prevent harm to the patient. The principle of justice, also referred to as social justice, addresses the equitable and appropriate distribution of resources [17].

## Results

Throughout the focus groups, older adults expressed their opinions, expectations, and concerns on an AI-based CDSS, which were organized into five categories: (1) data use, (2) patient-AI-physician interaction, (3) patient care, (4) resources, and (5) framework.

### Data Use

Participants emphasized that the quality of the CDSS, including its technical functionality, depends on an adequate data foundation, while recognizing the patient as an uncertain data source. They pointed out the risk of blindly trusting memory-based information provided by older patients who might forget, cheat, or unintentionally provide inaccurate information in a hospital environment perceived as unusual or stressful.

Most discussants highlighted the importance of protecting health-related patient data and were critical of current data management practices. While participants stressed that data protection measures would be required, they anticipated that security gaps would likely persist:

[The data security] is full of holes [...] sometimes. […] Much is impeded but the reality is sometimes bitterly different.

[Participant]

Data manipulation and hacking attacks were classified as high risk. The discussants emphasized concerns about targeted data alteration and about physicians unknowingly referring to the falsified data if such manipulation remained undetected.

In addition, concerns were raised about data access by third parties. Most participants highlighted the risk of data misuse, while a few pointed out that the level of concern would depend on the individual’s age and extent of misuse.

### Patient-AI-Physician Interaction

The discussants noted that older patients’ interactions with, and perspectives of, AI and the physician were influenced by individual personality traits. Three traits emerged from the discussions: (1) passive and trusting, (2) proactive, and (3) skeptical and experience-led (Table 1) .

**Table 1.** Representation of personality traits as influencing factors in the interactions and perspectives of older adults.

	Passive and trusting	Proactive	Skeptical and experience-led
Trust in physicians using AI^a^-based system	Complete trust in physician and health care system. Trust promotes acceptance of the use of any medical device.	Precondition: explanation of the system and involvement of the patient.	None: based on AI error rates and prior experiences of misdiagnosis.
Decision about using AI-based system	No involvement	Full involvement: right to demand use of AI-based system	Involvement: right to reject use of AI-based system
Participation (factor: cognitive capability)	None	Active participation of patient: entering their own data into the system	Gaining insights into visualizations as a basis for a discussion with the physician
Explainability of AI-based suggestions	Unnecessary	Highly important	Limitation: AI is not explainable (explainability is questionable when AI explains its own system)
Patient rights	Right not to know	Access right to the CDSS^b^ to add missing data	Reviewing AI-based results

a AI: artificial intelligence.

b CDSS: clinical decision support system.

The relationship between the physician and the AI was expected to evolve into mutual supervision: the AI would oversee the physician’s knowledge, while the physician would monitor the AI’s accuracy.

The severity of illness was identified as an impact factor, with increasing severity making patients more receptive to treatment suggestions. In particular, in cases of severe illness, patients tended to place greater trust in potentially effective treatment options and to question the physician if their treatment suggestion seemed less effective:

If I suffer from a severe illness and AI says, “If this and that, it will turn out well.” And my physician says “Um.” Whom do I trust in this case?

[Participant]

According to participants, a habituation effect of the CDSS would develop. In the long term, patients would either trust the AI more than the physician or not notice its use. At the same time, regular use of the CDSS would lead physicians to accept AI-based results as a given, place blind trust in the system, and thereby risk misdiagnosis.

While several discussants described the AI as providing a second opinion alongside the physician’s assessment, others characterized the AI and physician together as forming a single first medical opinion. In this context, participants noted the risk that the option of obtaining a true second opinion could be lost if different physicians using the same CDSS arrived at similar conclusions.

### Patient Care

Diagnosis and therapy provision were expected to accelerate, and the identification of rare and complex diseases was expected to become easier.

A few participants believed that the CDSS could help prevent overdiagnosis. However, others expressed concerns about the risk of overdiagnosis and overtreatment, which could lead to the prescription of more medication and prophylaxis programs, thereby increasing patients’ sense of illness.

The discussants also emphasized the risk of treatment suggestions being influenced by calculations of cost of the lifetime gained, the patient’s financial status and age, or third-party interests. The participants called for shared decision-making that accounts for the patient’s preferences, individual circumstances, and stage of life:

The limitation is that the final decision is not made by the AI but by the human.

[Participant]

### Resources

According to the discussants, language barriers could be reduced by providing medical professionals with guidance on patient-friendly communication and by offering staff from abroad information either in a widely spoken international language or in their native language.

Some discussants anticipated time savings through the reduction of complexity. However, others noted that such savings might depend on blind trust in the AI and reduced patient-physician communication.

Numerous participants also expressed concern about the potential loss of clinical intuition as a hallmark of good physicians and the inability to work without technical support:

I would have the concern that they push AI with the reason “I save employees, we save salaries, because we can just combine less qualified persons with the technology.” I think that only works well with good physicians.

[Participant]

### Framework

The hospital as an institution was expected to benefit from the CDSS through reduced patient length of stay and improved coordination of processes. However, discussants expressed concerns about a potential increase in patient numbers and a reduction in the time allocated for individual treatment. Furthermore, stress, lack of time, and convenience could lead physicians to blindly trust the CDSS:

The downside would perhaps be a transit operation. Each patient has five minutes, has only certain things to tell, to want and you press onto the computer and that is it.

[Participant]

Most of the participants insisted that the AI-based system should not be financed by the private sector, as a precaution during its implementation. This would help prevent profit-oriented influences on the CDSS in the sensitive field of older patient care.

## Discussion

**Figure 1.**
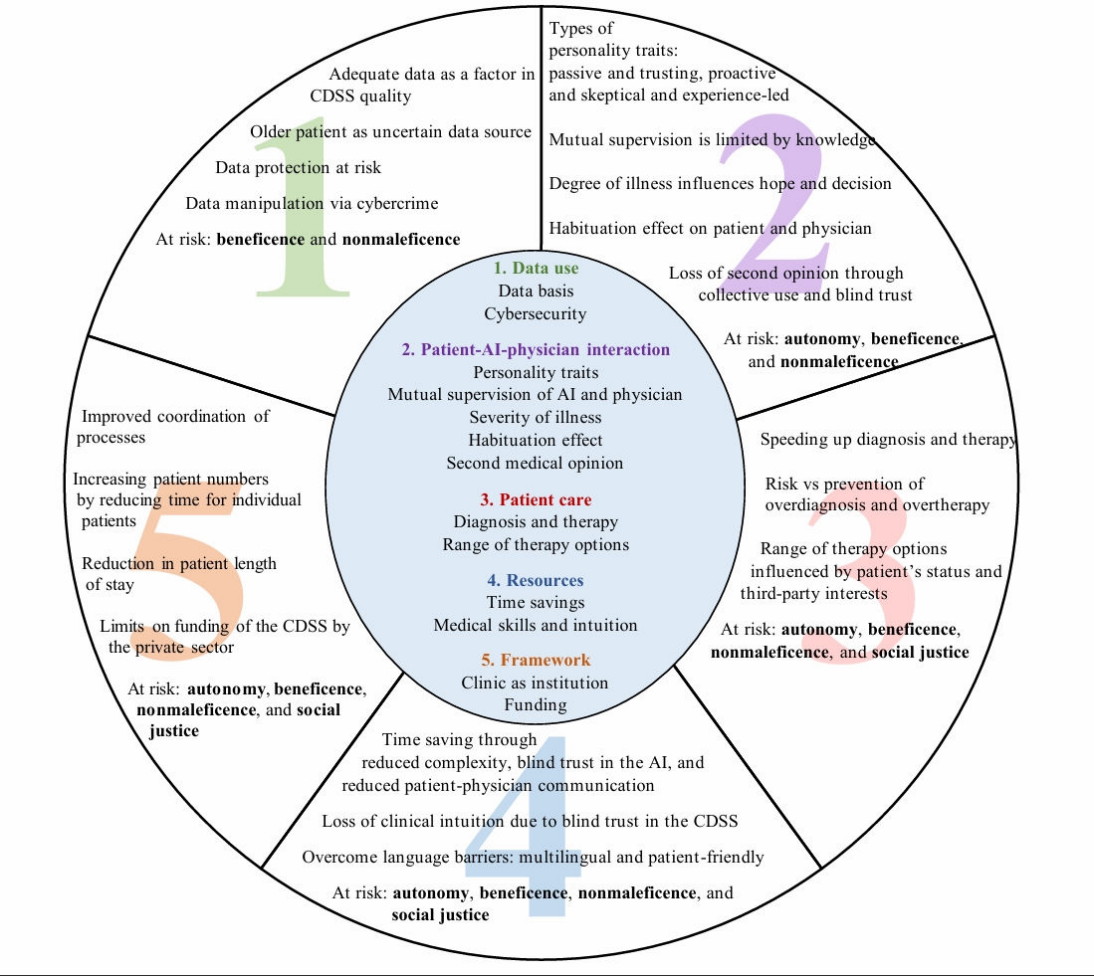
Representation of ethical challenges raised by older adults regarding the introduction of an artificial intelligence (AI)–based clinical decision support system (CDSS) in older patient care.

Ethical opportunities, concerns, and limitations of an AI-based CDSS, as identified by older adults, are discussed with reference to the 4 principles proposed by Beauchamp and Childress [17]: autonomy, beneficence, nonmaleficence, and social justice (Figure 1).

### Data Use

Participants described CDSS quality as dependent on an adequate data foundation and viewed the older patient as an uncertain data source. The completeness and accuracy of data were identified as the most relevant factors influencing system quality [18]. Low data quality, including insufficient or missing medical information, carries the risk of biased models being developed, which may lead to adverse results from the AI-based system [19]. However, self-reported data from older patients are considered less reliable [20], as they tend to forget or inaccurately recall information in hospital settings [20]. This uncertainty in the underlying data reduces system performance and quality, increasing the risk of incorrect medical suggestions and treatment. Consequently, patient harm from inappropriate treatment or diminished quality of medical care would jeopardize the principle of nonmaleficence.

Discussants expressed concern about cybersecurity. Cyberattacks are an increasing risk in the health care system [21]. Data that are altered, stolen, or manipulated [21] may go undetected [22] and could compromise CDSS recommendations. Medical decision-making based on false data puts patients at risk of receiving incorrect or inadequate treatment, thereby jeopardizing the principle of beneficence. In addition, the misuse of patients’ personal data, violations of privacy, and the implementation of incorrect treatment resulting from altered data further threaten the principle of nonmaleficence.

### Patient-AI-Physician Interaction

The personality traits of (1) passive and trusting, (2) proactive, and (3) skeptical and experience-led were identified as factors influencing older adults’ perspectives. Personality traits shape individual preferences [23], affect older patients’ perceptions of the CDSS [24], and influence expectations of the device.

The relationship between physician and AI was expected to develop into mutual supervision. Complementing each other’s knowledge and detecting incorrect evaluations [25] through mutual supervision could prevent incorrect treatment and simultaneously improve medical care. This relationship could arise from physicians supervising the AI by interpreting its results, while the AI minimizes the human errors of the physician [26]. The benefits that older patients experience from improvements in their medical care would support the principle of beneficence, whereas preventing patients from receiving incorrect medical treatment by minimizing human errors would strengthen the principle of nonmaleficence. However, mutual supervision remains imbalanced, given that the detection of AI-based errors is limited by the physician’s knowledge [27] and the lack of explainability of black-box systems [28]. In such cases, rather than mutual supervision, a paternalistic relationship could develop between the AI and the physician [28].

Furthermore, illness severity was described as an impact factor. Life-threatening diseases tend to increase patients’ hope for potential cures and create uncertainty when trusting a physician’s less effective treatment options. Transparent communication of AI-based results is therefore necessary to enable patients to make autonomous, informed decisions and to demonstrate the range of available treatment options. However, physicians do not necessarily want to communicate AI-based results transparently to their patients; they would rather withhold information [29]. Withholding medical information hinders shared decision-making [29] and undermines a trustful patient-physician relationship. Uncertainty in trust may cause patients to experience worry and avoid decision-making [30]; it may also reduce their ability to make rational treatment choices. Consequently, the consideration of patients’ individual preferences in medical decision-making, influenced by the severity of their illness, could be restricted, compromising the principle of autonomy.

Participants anticipated the development of a habituation effect. After becoming familiar with the application, patients might come to trust the AI more than the physician. Such distrust of the physician could lead patients to become skeptical of medical recommendations [31], thereby reinforcing uncertainty. Medical decision-making based on uncertainty and a general mistrust of physicians’ recommendations undermines patients’ independent decision-making, thereby jeopardizing the principle of autonomy. In addition, a general mistrust of medical recommendations could lead patients to decline beneficial treatment options, thus compromising the principle of beneficence. Physicians using the device regularly were also assumed to develop blind trust in the CDSS. Previous research has shown that habituation can lead users to take advantage of systems that permit unquestioned acceptance of suggestions, despite the high risks involved [32]. Blindly following CDSS suggestions that may not be accurate carries the risks of misdiagnosis and incorrect treatment, thereby jeopardizing the principle of nonmaleficence.

The discussants highlighted the risk of losing access to a genuine second medical opinion when different physicians using the same system reach similar conclusions. In this context, the combination of device and physician was described as a single first medical opinion. While a CDSS is designed to support physicians by providing a second medical evaluation, patients retain the option of consulting another physician for a second medical opinion [33]. However, if physicians collectively place blind trust in the CDSS and if similar systems generating similar results are used across hospitals, patients risk being deprived of a second medical opinion. Such circumstances would undermine patients’ independent consideration of medical options and preference-based decision-making, thereby conflicting with the principle of autonomy.

### Patient Care

The discussants emphasized that the CDSS would speed up diagnosis, particularly in rare and complex diseases, as well as facilitate the provision of treatment. The medical use of AI enables rapid analysis of patient data, leading to a reduction in the time required for diagnosis [34]. Early diagnosis in older patients provides a basis for timely initiation of treatment, lowering the risk of impairment and secondary complications [35]. As a result, overall medical care would improve. Prompt diagnosis may also enhance the healing process of older patients, thereby supporting the principle of beneficence. Furthermore, the prevention and decreased risk of medical complications through timely treatment provision aligns with the principle of nonmaleficence.

Several participants expressed concern that the CDSS could contribute to overdiagnosis and overtreatment, causing patients to feel increasingly ill, while others suggested that it could prevent these problems. Previous studies with AI-based screening devices have shown that AI can accurately detect cell changes that are not necessarily pathological but nevertheless result in overtreatment [36]. Overdiagnosis exposes patients to unnecessary medical treatments and the associated risk of harmful side effects [37], thereby conflicting with the principle of nonmaleficence. Overdiagnosis can also heighten patients’ perception of illness, and when combined with treatment that offers no medical benefit, it further undermines the principle of beneficence. In addition, the preventable use of already limited resources fosters an imbalanced distribution of medical resources, conflicting with the principle of social justice. Conversely, if the CDSS helps prevent overdiagnosis and overtreatment, it could improve the provision of essential treatment, supporting the principle of beneficence. Preventing insufficient treatment in older patients and the resulting consequences would also reinforce the principle of nonmaleficence.

Participants expressed major concerns about treatment options being influenced by financial considerations and third-party interests. Security gaps and unauthorized access to data or algorithms could enable alterations to the AI-based system [38], thereby influencing CDSS outcomes. Restricting treatment options to serve profit-oriented interests of third parties could result in less effective treatment options for older patients. Such restrictions reduce older patients’ capacity for informed decision-making and jeopardize the principle of autonomy. Limiting treatment options on the basis of third-party or financial interests carries the risk of excluding effective treatments, conflicting with the principle of beneficence. Moreover, narrowing treatment options in ways that compromise patient outcomes would undermine the principle of nonmaleficence.

### Resources

Participants expected time savings through reduced complexity, blind trust in the CDSS, and avoidance of extensive patient-physician communication. However, given the limited accuracy of a CDSS [39], physicians must review the AI-generated results individually to reduce the risk of incorrect treatment. Understanding the individual preferences and concerns of older patients requires time-intensive conversation [40] and is fundamental for decision-making. A lack of communication regarding the patient’s preferences could lead to medical decision-making that does not sufficiently take these preferences into account, thereby jeopardizing the principle of autonomy. Relying blindly on the CDSS without examining the results for false implications could harm patient outcomes and compromise the principle of nonmaleficence. Together, blind trust in the CDSS and reduced patient-physician communication could undermine patient-centered medical care and treatment and, in turn, jeopardize the principle of beneficence.

The discussants expected the CDSS to help medical staff overcome language barriers. Effective communication between patients and medical professionals is necessary to identify individual concerns and to build trust [41]. Language barriers may cause patients to avoid follow-up appointments [42], leading to undetected postoperative complications. Poor medical care and worse outcomes resulting from communication difficulties [43] could be prevented by reducing language-related barriers. A multilingual, patient-centered use of the AI-based CDSS could therefore improve patient care and support the principle of beneficence. Improved communication about side effects, treatment risks, and patient concerns could support the principle of nonmaleficence. In addition, language support for medical staff from abroad can save time and human resources while laying the foundation for equitable medical treatment regardless of the language skills of patients or staff, thereby strengthening the principle of social justice.

Medical professionals who regularly use the CDSS were expected to lose clinical intuition and develop a dependency on technical support. Some researchers suggest that the CDSS could enhance medical skills by allowing physicians to learn from its results [44]. However, blind trust in the system may instead reduce medical skills and foster dependency [44]. As a result, the accuracy of medical treatment and thus the protection of patient integrity could become dependent on technology, jeopardizing the principle of beneficence. Moreover, the ability of physicians to verify suggested results and prevent incorrect treatment might no longer be ensured, thereby compromising the principle of nonmaleficence.

### Framework

According to participants, hospitals would benefit from the CDSS through better coordination of processes, shorter patient stays, and reduced time spent on individual patient care, thereby enabling treatment of more patients. The shortest possible perioperative stay is financially the most profitable [45]. Minimizing length of stay while increasing patient volume enhances hospital revenue and saves resources. In addition, a higher number of patients could receive treatment, supporting the principle of social justice. However, a balance must be maintained between the needs of individual patients and those of the broader population. Older patients with multiple comorbidities, in particular, face a higher risk of mortality after premature discharge [46]. Profit-driven discharge of patients before medical necessity increases the risk of delayed complication detection and increased mortality in older patients, thereby jeopardizing the principle of nonmaleficence. Delayed treatment of postoperative complications could further expose patients to more severe consequences and subsequently compromise the principle of beneficence.

The discussants were emphatic about the need to restrict private-sector funding of the CDSS to prevent profit-oriented influence. As the algorithms and underlying data of a CDSS are adjustable [38], treatment options could be altered to favor sponsors. This would restrict the consideration of patients’ individual preferences in medical decision-making and thereby jeopardize the principle of autonomy. Limiting treatment options for financial gain could also result in withholding effective treatments, undermining the principle of beneficence. Moreover, biased treatment suggestions could expose patients to incorrect medical care, compromising the principle of nonmaleficence.

### Conclusions

This research indicates that older adults anticipate both opportunities and ethical challenges in data use, the patient-AI-physician interaction, patient care, resources, and framework when an AI-based CDSS is introduced in older patient care.

To address the ethical concerns raised by the limited number of potential patients in this qualitative research, we recommend allowing sufficient time for use of the CDSS to account for individual patient concerns and preferences. Furthermore, individualized review of AI-generated suggestions and avoidance of private sponsorship are recommended to reduce the risk of less effective medical treatments.

This research is limited by the fact that the participants lived in southern Germany and were privileged in having access to the internet and an academic network. Given the qualitative design, their subjective opinions do not represent average experiences and cannot be generalized to the global older population.

To further analyze the concerns of older adults, consideration of gender and differentiation by age ranges (eg, 65-74, 75-84, and ≥85 years) is recommended. On the basis of our small sample of participants from a single region in Germany, we recommend continuing the research with larger samples involving transregional and transnational participants to examine the scope and generalizability of our findings.

## Abbreviations

AI: artificial intelligence
CDSS: clinical decision support system

## References

[1] Life expectancy at birth, total (years). World Bank Group.

[2] How university medical centers can best position themselves for changes across society. Charité – Universitätsmedizin Berlin.

[3] Share of population that are aged 65 years and older in european countries in 2023. Statista.

[4] Loyd C, Markland AD, Zhang Y (2020). Prevalence of hospital-associated disability in older adults: a meta-analysis. J Am Med Dir Assoc.

[5] Van Grootven B, Flamaing J, Dierckx de Casterlé B (2017). Effectiveness of in-hospital geriatric co-management: a systematic review and meta-analysis. Age Ageing.

[6] Rapp K, Becker C, Todd C (2020). The association between orthogeriatric co-management and mortality following hip fracture. Dtsch Arztebl Int.

[7] Barnett K, Mercer SW, Norbury M, Watt G, Wyke S, Guthrie B (2012). Epidemiology of multimorbidity and implications for health care, research, and medical education: a cross-sectional study. The Lancet.

[8] Aubert CE, Streit S, Da Costa BR (2016). Polypharmacy and specific comorbidities in university primary care settings. Eur J Intern Med.

[9] Aravazhi PS, Gunasekaran P, Benjamin NZ (2025). The integration of artificial intelligence into clinical medicine: trends, challenges, and future directions. Dis Mon.

[10] Damoiseaux-Volman BA, van der Velde N, Ruige SG, Romijn JA, Abu-Hanna A, Medlock S (2021). Effect of interventions with a clinical decision support system for hospitalized older patients: systematic review mapping implementation and design factors. JMIR Med Inform.

[11] Leinert C, Fotteler M, Kocar TD (2023). Supporting SURgery with GEriatric co-management and AI (SURGE-Ahead): a study protocol for the development of a digital geriatrician. PLoS ONE.

[12] Skuban-Eiseler T, Orzechowski M, Denkinger M, Kocar TD, Leinert C, Steger F (2023). Artificial intelligence-based clinical decision support systems in geriatrics: an ethical analysis. J Am Med Dir Assoc.

[13] Krueger RA, Casey MA (2015). Focus Groups- A Practical Guide for Applied Research.

[14] Hennink MM (2014). Focus Group Discussions- Understanding Qualitative Research.

[15] Mayring P (2015). Qualitative Inhaltsanalyse- Grundlagen Und Techniken [Book in German].

[16] Braun V, Clarke V (2006). Using thematic analysis in psychology. Qual Res Psychol.

[17] Beauchamp T, Childress J (2019). Principles of Biomedical Ethics.

[18] Budach L, Feuerpfeil M, Ihde N (2022). The effects of data quality on machine learning performance. arXiv.

[19] Carini C, Seyhan AA (2024). Tribulations and future opportunities for artificial intelligence in precision medicine. J Transl Med.

[20] Savioli G, Ceresa IF, Giordano M (2021). The reliability of anamnestic data in the management of clostridium tetani infection in elderly. Front Med (Lausanne).

[21] Wasserman L, Wasserman Y (2022). Hospital cybersecurity risks and gaps: review (for the non-cyber professional). Front Digit Health.

[22] Mirsky Y, Mahler T, Shelef I (2019). CT-GAN: malicious tampering of 3D medical imagery using deep learning. arXiv.

[23] Flynn KE, Smith MA (2007). Personality and health care decision-making style. J Gerontol B Psychol Sci Soc Sci.

[24] Redelmeier DA, Najeeb U, Etchells EE (2021). Understanding patient personality in medical care: five-factor model. J Gen Intern Med.

[25] Kempt H, Heilinger JC, Nagel SK (2023). “I’m afraid I can’t let you do that, Doctor”: meaningful disagreements with AI in medical contexts. AI & Soc.

[26] Göndöcs D, Dörfler V (2024). AI in medical diagnosis: AI prediction & human judgment. Artif Intell Med.

[27] Richard A, Mayag B, Talbot F (2020). What does it mean to provide decision support to a responsible and competent expert?: The case of diagnostic decision support systems. EURO J Decis Process.

[28] Xu H, Shuttleworth KMJ (2024). Medical artificial intelligence and the black box problem: a view based on the ethical principle of “do no harm”. Intelligent Medicine.

[29] Lorenzini G, Arbelaez Ossa L, Shaw DM, Elger BS (2023). Artificial intelligence and the doctor-patient relationship expanding the paradigm of shared decision making. Bioethics.

[30] Hillen MA, Gutheil CM, Strout TD, Smets EMA, Han PKJ (2017). Tolerance of uncertainty: conceptual analysis, integrative model, and implications for healthcare. Soc Sci Med.

[31] Williamson LD, Thompson KM, Ledford CJW (2022). Trust takes two…. J Am Board Fam Med.

[32] Hoesterey S, Onnasch L (2023). The effect of risk on trust attitude and trust behavior in interaction with information and decision automation. Cogn Tech Work.

[33] Isaacs D (2023). The second opinion. J Paediatrics Child Health.

[34] S Alshuhri M, Al-Musawi SG, Al-Alwany AA (2024). Artificial intelligence in cancer diagnosis: opportunities and challenges. Pathol Res Pract.

[35] Heppner HJ, Haitham H (2022). Intensive care of geriatric patients-a thin line between under- and overtreatment. Wien Med Wochenschr.

[36] Mori Y, Misawa M, Bernal J (2022). Artificial intelligence for disease diagnosis: the criterion standard challenge. Gastrointest Endosc.

[37] Gupta P, Gupta M, Koul N (2020). Overdiagnosis and overtreatment; how to deal with too much medicine. J Family Med Prim Care.

[38] Brown S, Davidovic J, Hasan A (2021). The algorithm audit: scoring the algorithms that score us. Big Data Soc.

[39] Gong Y, Min H, Jing X, Yu P (2024). Challenges and opportunities of artificial intelligence in CDSS and patient safety. Stud Health Technol Inform.

[40] Rostoft S, van den Bos F, Pedersen R, Hamaker ME (2021). Shared decision-making in older patients with cancer - what does the patient want?. J Geriatr Oncol.

[41] Kwame A, Petrucka PM (2021). A literature-based study of patient-centered care and communication in nurse-patient interactions: barriers, facilitators, and the way forward. BMC Nurs.

[42] Chandrashekar P, Zhang R, Leung M, Jain SH (2022). Impact of patient-physician language concordance on healthcare utilization. J Gen Intern Med.

[43] Luan-Erfe BM, Erfe JM, DeCaria B, Okocha O (2023). Limited English proficiency and perioperative patient-centered outcomes: a systematic review. Anesth Analg.

[44] Sutton RT, Pincock D, Baumgart DC, Sadowski DC, Fedorak RN, Kroeker KI (2020). An overview of clinical decision support systems: benefits, risks, and strategies for success. NPJ Digit Med.

[45] Hashmi SA, Raja MHR, Arif A, Naseem Z, Pal KMB, Pal KMI (2024). Reducing post-operative length of stay, is it worth the effort?. World J Surg.

[46] Aasbrenn M, Christiansen CF, Esen BÖ, Suetta C, Nielsen FE (2021). Mortality of older acutely admitted medical patients after early discharge from emergency departments: a nationwide cohort study. BMC Geriatr.

